# Willingness to pay for social health insurance among public civil servants: A cross-sectional study in Dessie City Administration, North-East Ethiopia

**DOI:** 10.3389/fpubh.2022.920502

**Published:** 2022-07-19

**Authors:** Eshetie Meseret Amilaku, Fasil Walelign Fentaye, Asnakew Molla Mekonen, Ewunetie Mekashaw Bayked

**Affiliations:** ^1^Department of Nursing, Dessie Comprehensive Specialized Hospital, Dessie, Ethiopia; ^2^Department of Health Systems and Management, School of Public Health, College of Medicine and Health Sciences, Wollo University, Dessie, Ethiopia; ^3^Department of Pharmacy, College of Medicine and Health Sciences, Wollo University, Dessie, Ethiopia

**Keywords:** willingness to pay, public civil servants, Ethiopia, factors, social health insurance

## Abstract

**Background:**

The readiness to pay for health insurance has an impact on universal health care. This study investigated the willingness of public civil servants in Dessie City Administration, North-East Ethiopia, to pay for social health insurance and associated factors.

**Methods:**

From April to May 2021, a cross-sectional study was undertaken. The sectors from which the samples were proportionately allocated were chosen using a multistage sampling procedure. Using their payroll list as a sampling frame, simple random sampling was done to recruit them. A semi-structured self-administered questionnaire was used to collect data. Epi Data version 3.1 was used to enter, code, and clean the data, which was then exported to SPSS version 23 for analysis. To determine relationships, bi-variable and multivariable analyses were utilized. Candidates for multivariable analysis were variables with a *p*-value ≤ 0.3 in bi-variable analysis. AOR with a 95% CI was used to determine the strength and direction of association. Statistical significance was defined at *p*-value < 0.05.

**Results:**

A total of 796 employees took part in the study, with a 94.42% response rate. Overall, 29.60% of them were willing to pay for the scheme (95% CI: 26.4, 33%). The decision to pay was influenced by favorable perception (AOR = 2.43, 95% CI: 1.72, 3.44), household income < Birr 5,000 (AOR = 0.26, 95% CI: 0.14, 0.48), acute illness (AOR = 0.48, 95% CI: 0.32, 0.72), bachelor level education (AOR = 0.44, 95% CI: 0.28, 0.70), master and above level education (AOR = 0.26, 95% CI: 0.14, 0.49), and age 25–29 years (AOR = 1.97, 95 % CI: 1.08, 3.57).

**Conclusion:**

The willingness of the civil servants to pay for social health insurance was low, and it was influenced by their attitudes toward the scheme, the occurrence of severe illness, income level, educational status, and age.

## Introduction

Universal health care (UHC) is the central objective of Sustainable Development Goal (SDG) 3 (1). UHC is firmly based on the 1948 World Health Organization (WHO) Constitution (2). Social health insurance (SHI) is one of the sustainable mechanisms to achieve UHC, mainly through cost sharing between beneficiaries and government (3). It is critically depend on the utmost amount of money that an individual is willing to forego; i.e., the willingness-to-pay (WTP) (4). Though various SHI programs have been developed in Sub-Saharan African (SSA) nations (5), their scope and provenance are hazy (6). In SSA, the main sources of health care financing (HCF) are government budgets (7, 8), general revenues and direct out-of-pocket (OOP) payments (9). The OOP expenditure has known to be the major catastrophe. It impacts the overall aspect of healthcare utilization (10); especially in countries like Ethiopia where healthcare costs are very high (11).

Since the Alma-Ata Declaration on Primary Health Care (PHC) in 1978, Ethiopia has made several attempts to attain UHC (12). The healthcare sector of the country is financed by loans and donations (46.8%), the Government (16.5%), OOP payments (35.8%), and other sources (0.9%) (12). As mentioned here, the OOP payment is still quite substantial, which dictates that health financing remains a major challenge to UHC (7, 13). To overcome this challenge, the country has introduced SHI and community-based health insurance (CBHI) to cover 10.46 and 84% of the population, respectively (14). However, SHI has been postponed multiple times due to substantial resistance for the WTP from public servants, despite the fact that it was meant to be fully implemented by 2014 (12). However, the reasons may vary from person to person and region to region (15, 16). Accordingly, this study aimed to investigate the WTP for SHI and associated factors among public civil servants in Dessie City Administration (DCA), North-East Ethiopia.

## Materials and Methods

### Study design and setting

A cross-sectional study was conducted from April to May 2021 in DCA, North-East Ethiopia from April to May 2021. The city is largest urban center in North-East Ethiopia and is characterized by various socio-demographic features (17) (Table 1).

**Table 1:** Description of DCA (17).

Significance	Largest urban center in North-East Ethiopia
Population	Town (209,226), Rural (35,903), Total (245,129)
Ethnic groups	Amhara (94.89%), Tigre (3.79%); Others (0.67%)
Religions	Islam (58.62%), Orthodox (39.92), Protestant (1.15%), and Others (0.31%)
Language	Amharic (95%), Tigrigna (4%); Others (1%)
Economy	Economically active (50.85%), Economically inactive (49.15%)
Employment	Employed (88%), Unemployed (12%)

### Participants and sample

The source population was all public servants working in government sectors of DCA. The study population consisted of all public servants working in selected sectors, while the study units were all individual level public servants in randomly selected specific sectors, from whom the samples were obtained. All permanently employed public civil servants were included. Employees who refused to participate, were critically ill and not available at the time of data collection were excluded. The sample size was calculated using the single population proportion formula with an assumption of the proportion of the WTP for SHI (47%) in Wolayita Sodo (15), 5% margin of error (d), 95% CI, 2 design effect (deff) and 10% non-response rate. Accordingly:


(1) $$\begin{array}{r} n=deff\frac{Z^{2}\;\left(p\right)\left(1-p\right)}{d^{2}}\;=2\frac{1.96^{2}\;\left(0.47\right)\left(0.53\right)}{0.05^{2}}\;=\;766 \end{array}$$

The sample size was increased to 843 by adding 10% non-response (766 × 0.1 = 77). As a result, the final sample size was determined to be 843. Then, out of 42 total governmental institutions, 12 institutions, with a total of 1,295 civil servant, were randomly selected and the samples were proportionately allocated (Table 2). Then, using their payroll list as a sampling frame, they were chosen using a simple random sampling procedure.

**Table 2:** The sampling procedure among governmental institutions in DCA, 2021.

**Institution**	**No. of civil servants**	**Samples allocated**
Education department	170	111
Food security department	17	11
Health department	155	101
Department of civil service	38	25
Culture, tourism and sport department	76	49
Worker and social agency department	24	16
Department of female and children's affairs	24	16
Department of agriculture	85	55
Civil servants at Hote Sub-City	247	161
City construction and development office	59	38
Department of finance and economy	53	34
Civil servants at Segno Gebaya Sub-City	347	226
Total	1,295	843

### Data collection procedures

A self-administered questionnaire was used to collect data, which was prepared based on previous literatures and adjusted as needed (15, 18–23). It was divided into five sections: sociodemographic factors, SHI knowledge, SHI perception, health and health-related issues, and WTP (readiness to pay) for SHI. The amount of contribution to the SHI plan for an employee and employer is 3% of the member's gross wage, according to Council of Ministers Regulation No. 271/2012 (24). So, the data collectors informed the participants that the monetary value in determining the WTP was 3% of their incomes. As a result, since the participants have been notified of the amount to be paid for the scheme (3% of their gross salary), we did not use a monetary range to determine the WTP.

- **Socio-demographic features:** The questionnaire contained socio-demographic information such as gender, age, religion, marital status, educational level, type of profession, years of service, monthly individual and household income and source, and family size.
- **SHI knowledge:** The tool included general concepts such as the conception of SHI information; the source of that information; who might be covered by SHI; the need to pay premiums for SHI due to anticipated health problems; the costs to be covered by the SHI program; and whether to claim or not to claim, as well as the fate of the to be paid premium. Civil servants who scored above the median on the questions were judged to have “good knowledge,” while those who scored below the median were considered to have “poor knowledge.”
- **SHI perception:** Ideas including the safety of the payment method, perceived quality of health care within SHI, adequacy of SHI benefit packages, and the state of financial instability in government institutions were appraised in order to study what the participants thought about SHI. Civil servants who scored above the median on the questions were judged to have “good knowledge,” while those who scored below the median were considered to have “poor knowledge.” The civil servants with scores above the median were deemed to have “good perception,” while those with scores below the median were deemed to have “poor perception.”
- **Health and health related issues:** Issues such as the presence of a chronic condition, any recent illness episode (morbidity), and the number of family members afflicted; whether or not healthcare was sought; the reason for seeking healthcare or not; the total health care cost of the household in the previous 6 months; how and by whom was the cost of that health care covered; the level of satisfaction with the service and costs; the quality of service; the feeling of paying OOP; and whether borrowing was a matter or not were all assessed.
- **WTP for SHI:** To determine the participants' interests in contributing to SHI, the extents of their WTP for SHI from monthly paychecks were leveled and assessed in proportions as follows. The WTP if the premium is 2, 3, or 4%, their maximum interest to pay per month, 8, 9, or 10%, and the maximum amount to pay if inflation or other uncertainties exist were all evaluated.

The lottery approach (simple random sampling) was used to choose the participants based on their proportionate sample size. Six data collectors, all of whom were BSc nurses with prior data gathering expertise, took part in the study. The data were collected at the participants' workplaces.

### Data processing and analysis

Epi Data version 3.1 was used to enter, code, check, and clean the data, which was then exported to SPSS version 23 for analysis. Frequency tables and graphs were used to display the descriptive statistics. The bi-variable binary logistic regression analysis was used to determine the relationship between each independent variable and the outcome variable, and those variables with a *p*-value < 0.3 in the bi-variable analysis were entered into the multiple logistic regression model to control for all potential confounders and identify the independent factors of the outcome variable. Since the selection of a higher significance level has the disadvantages that some unimportant variables may be included in the model, we chose a cut-off *p*-value of < 0.3 to allow some variables to exit the selection process (25). Standard error was used to check for multi-collinearity between independent variables. The model fitness was determined using the Hosmer-Lemeshow test (*p*-value = 0.225). The association between categorical variables was examined using Pearson's chi-square test. The variables with a *p*-value < 0.05 were considered statistically significant in the final model. The strength and direction of association were calculated using the adjusted odds ratio (AOR) and the 95% CI. Because of missing data, 47 questionnaires were omitted from the final analysis.

### Data quality control

The questionnaire was written in English and then translated into Amharic then back to English for consistency. In a similar setting, the questionnaire was pre-tested in 10% of participants (Kombolcha City Administration). The survey took place over the course of 2 months (April–May 2021). Two investigators (EMA and EMB) and two supervisors (FWF and AMM) made up the team. The investigators (EMA and EMB) provided the data collectors with a half-day of theoretical and field instruction. They were constantly monitored and followed up on. On a daily basis, the completed surveys were verified for clarity and completeness.

## Results

### Socio-demographic descriptions

A total of 796 people from 12 different sectors took part in this study, with a response rate of 94.42%. Participants were 36 (±4) years old on average, with 246 (63.2%) being in the age range of 25–29 years. Five hundred nine (63.9%) participants had a bachelor's degree, while 139 (17.5%) had a master's degree or above. Males made up 465 (58.4%) of those who took part in the study. More than half of the respondents (55.7%) were orthodox Christians, while 39.5% were Muslims (Table 3).

**Table 3:** Sociodemographic descriptions of public civil servants in DCA, North-East Ethiopia (*n* = 796), May 2021.

**Variables**	**Category**	***N*** **(%)**	**X**^2^ **(Chi-square)**	**p-value**
Age	<25 years	33 (4.1)	8.73	0.068
	25–29 years	163 (20.5)		
	30–34 years	222 (27.9)		
	34–39 years	132 (16.6)		
	>39 years	246 (30.9)		
Sex of participants	Male	465 (58.4)	0.00	0.98
	Female	331 (41.6)		
Marital status	Married	583 (73.2)	3.32	0.19
	Single	170 (21.4)		
	Others**	43 (5.4)		
Educational status	MSc and above	139 (17.5)	7.03	0.03
	Degree	509 (63.9)		
	Up to diploma	148 (18.6)		
Religion	Orthodox	443 (55.7)	2.47	0.29
	Muslim	315 (39.5)		
	Other*	38 (4.8)		
Profession	Health professional	117 (14.7)	6.27	0.28
	Teacher	168 (21.1)		
	Engineer	68 (8.5)		
	Management and accounting	143 (18.0)		
	HIT	71 (8.9)		
	Other***	219 (27.5)		
Family size	1–2	118 (14.8)	2.95	0.23
	3–4	401 (50.4)		
	>4	267 (33.5)		
Monthly net household income	≤ 5,000	186 (23.4)	16.84	0.00
	5,001–10,000	363 (45.6)		
	≥10,001	208 (26.1)		

^*^Protestant and Catholic, ^**^divorced and widowed, ^***^Agriculture, natural, and human resource and statistics.

### Knowledge, perception, and health status

**Figure 1:**
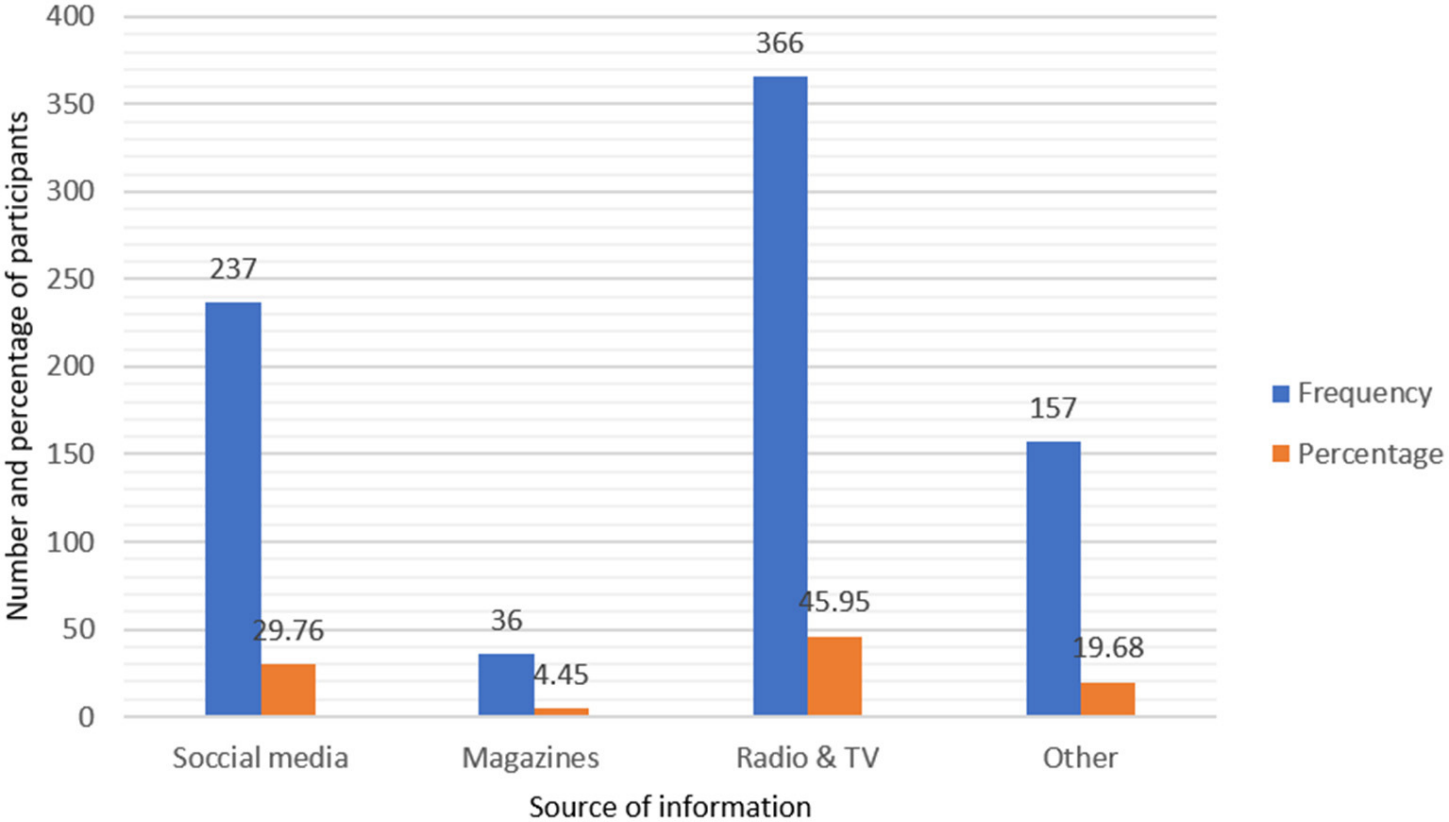
Public civil servants' source of information regarding SHI in DCA, North-East Ethiopia (*n* = 796), May 2021.

Five hundred eight (63.8%) of the participants had good understanding of social health insurance, whereas 360 (45.2%) had a positive impression of it (Table 4). The Chi-square result revealed that the participants' knowledge was associated to their WTP for SHI. In the regression analysis, however, it was not a significant factor. For the civil servants, broadcast media was the primary source of information on SHI. Three hundred sixty-six (45.95%) of the participants learned about SHI through radio and television broadcasts. Social media was another key source of information, with 29.76% of the participants using it (Figure 1). About 473 (59.4%) of the total respondents' homes had acute illness in the previous 6 months, while 176 (22.1%) had chronic disease (Table 4). About 326 (71.6%) of those who experienced acute illness in the previous 6 months had one ill person in their house, while the rest had two to four ill persons. About 451 (95.36%) of the total households with persons having acute illness received treatment on time. Those who did not receive treatment (45.5%) mention financial reasons as major stumbling block, and 54.5% believed their illness would be self-limited. With regards to service effectiveness and low cost, around 62.8% of them prefer treatment from government hospitals and health centers. About 53.9% were satisfied with the healthcare service. About 31.6% of respondents got treatment at a private health facility. Eighty-five percentage of those receiving treatment had not borrowed money.

**Table 4:** Knowledge and Illness related factors of public civil servants in DCA, North-East Ethiopia (*n* = 796), May 2021.

**Variables**	**Category**	***N*** **(%)**	**X**^2^ **(Chi-square)**	***p*** **-value**
Knowledge about SHI	Poor	288 (36.2)	4.68	0.03
	Good	508 (63.8)		
Perception related to SHI	Good	360 (45.2)	22.27	0.00
	Poor	436 (54.8)		
Acute illness within the last 6 months	Yes	473 (59.4)	30.19	0.00
	No	323 (40.6)		
Chronic illness within the last6 months	Yes	176 (22.1)	4.09	0.043
	No	620 (77.9)		

### Willingness to pay

**Figure 2:**
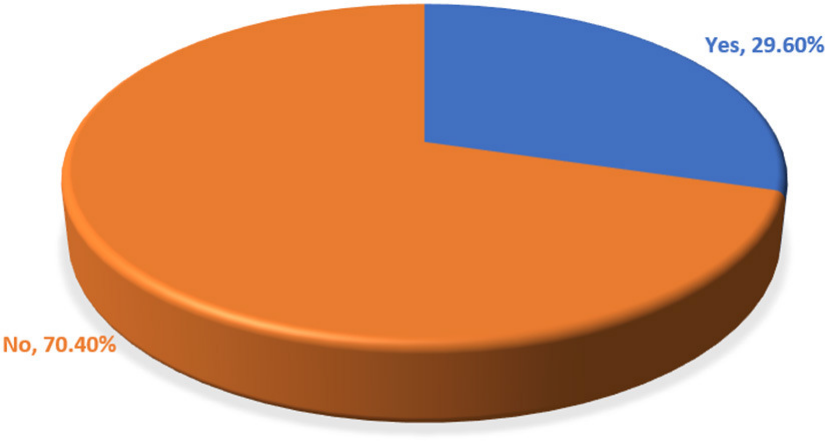
The overall proportion of the WTP for SHI among public civil servants in DCA, North-East Ethiopia (*n* = 796), May 2021.

**Figure 3:**
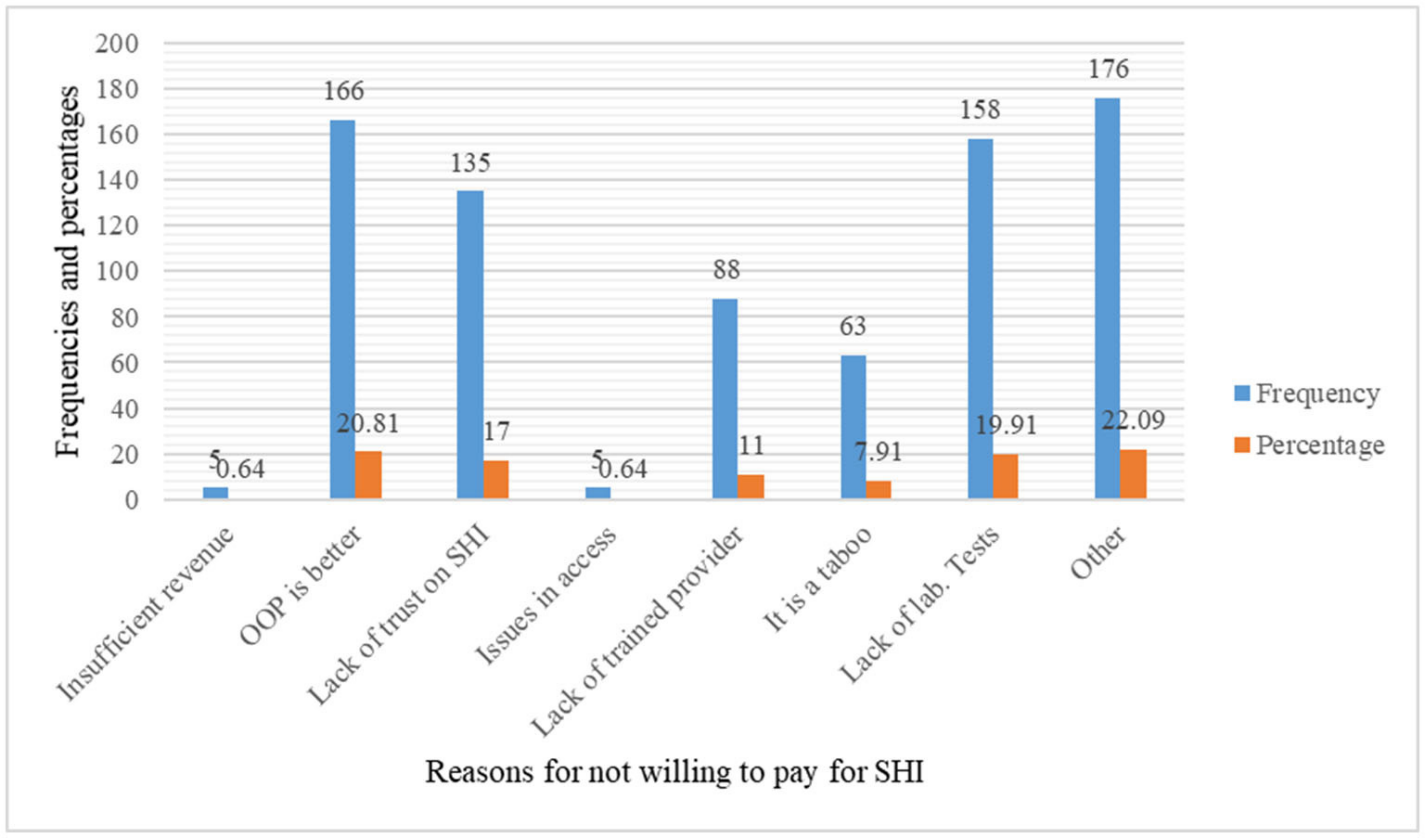
The reasons for not WTP for SHI among public civil servants at DCA, North-East Ethiopia (*n* = 796), May 2021.

The starting bid of participant's ability of the WTP for SHI is described below in Table 5. The aggregate proportion of the WTP for SHI in DCA by public civil personnel was 29.6% (95% CI: 26.4, 33.0) (Figure 2). The primary reasons for the WTP for SHI were to receive free services during illness (43.7%), to support others (26.3%), for security and peace of mind during illness (18.7%), and to deal with ailments that occur often (to face frequently occurred illnesses) (8%). On the other hand, the employees were not WTP for a variety of reasons (Figure 3). Inadequacy of salary, belief that OOP is preferable, lack of faith in SHI, issues with access to health inputs, perceptions of a lack of skilled providers, belief that it is taboo, and perceptions of a lack of laboratory tests are just a few of them.

**Table 5:** Summary statistics to double-bounded dichotomous choice questions with 3% starting bid on the WTP for SHI at DCA, North-East Ethiopia (*n* = 796), May 2021.

**Proposed bids**	**Yes**, ***N*** **(%)**	**No**, ***N*** **(%)**
WTP	493 (61.9%)	303 (38.1%)
WTP for the 1st bid	139 (21.4%)	354 (78.6%)
WTP for the 2nd lower bid	194 (30%)	453 (70%)
WTP for the 2nd higher bid	42 (8.2%)	451 (91.7%)

### Factors associated with the willingness to pay

In multi-variable analysis, eleven variables (age, marital status, educational status, type of profession, religion, family size, household income, perception of SHI, knowledge of SHI, acute and chronic disease) with *P*-values < 0.3 were taken into account in bi-variable logistic regression. Age, educational status, net family income, perception of SHI, and acute illness were revealed to have significant statistical associations with WTP for SHI in multivariable analysis.

The odds of WTP for SHI were about 2 times greater for persons aged 25–29 years compared to those aged 40 years or more (AOR = 1.96, 95% CI: 1.08, 3.56). WTP for SHI was 2.4 times greater for persons with positive perception of SHI compared to those with poor perception (AOR = 2.43, 95% CI: 1.72, 3.44). Individuals with a history of acute illness in the previous 6 months were 0.52 times less likely than their counterparts to be willing to pay for SHI (AOR = 0.48, 95% CI: 0.32, 0.72). The analysis also revealed that people with an MSc or higher education were 0.74 times less likely to pay for SHI than those with a diploma or less (AOR = 0.26, 95% CI: 0.14, 0.49). Similarly, participants with a bachelor's degree were 0.56 times less likely to pay for SHI than those with a diploma or less (AOR = 0.44, 95% CI: 0.28, 0.70). Finally, the multi-variable analysis revealed that individuals with a household income of <5,001 were 0.74 times less likely to pay for SHI than those with a household income of more than 10,000 (AOR = 0.26, 95% CI: 0.14, 0.48) (Table 6).

**Table 6:** Bi-variable and multi-variable logistic regression for WTP for SHI among public civil servants in DCA, North-East Ethiopia (*n* = 796), May 2021.

**Variables**	**Category**	**WTP for SHI**		**COR (95% CI)**	**AOR (95% CI)**
		**Yes**	**No**		
Age	<24 years	3	22	0.34 (0.098, 1.16)	0.34 (0.08, 1.37)
	25–29 years	58	105	1.36 (0.89, 2.08)	1.97 (1.08, 3.57) *
	30–34 years	58	164	0.87 (0.58, 1.31)	0.89 (0.54, 1.5)
	35–39 years	44	88	1.23 (0.78, 1.94)	1.05 (0.62, 1.78)
	>39 years	71	175	1	1
Educational status	MSc and above	29	110	0.50 (0.30, 0.83)	0.258 (0.14, 0.49) *
	Degree	156	353	0.84 (0.57, 1.24)	0.439 (0.28, 0.7) *
	Diploma and lower	51	97	1	1
Marital status	Married	182	401	1	1
	Single	44	125	1.72 (0.81, 3.65)	0.796 (0.46, 1.38)
	Others	9	34	1.33 (0.59, 2.99)	0.609 (0.27, 1.40)
Religion	Orthodox	124	319	1	1
	Muslim	96	218	1.13 (0.83, 1.56)	1.056 (0.73, 1.51)
	Other	15	23	1.68 (0.85, 3.32)	1.621 (0.74, 3.53)
Profession	Teacher	40	128	1	1
	Management and accounting	48	95	1.62 (0.98, 2.66)	1.67 (0.94, 2.98)
	Engineer	22	46	1.53 (0.82, 2.84)	1.55 (0.75, 3.21)
	Health	41	76	1.73 (1.03, 2.90)	0.89 (0.48, 1.66)
	HIT	18	53	1.09 (0.57, 2.07)	1.48 (0.68, 3.20)
	Other	66	153	1.38 (0.87, 2.18)	1.41 (0.83, 2.41)
Net household income	≤ 5,000	35	151	0.39 (0.24, 0.61)	0.26 (0.14, 0.48)*
	5,001–10,000	112	251	0.74 (0.52, 1.06)	0.66 (0.43, 1.00)
	≥10,001	78	130	1	1
Perception toward SHI	Good	137	223	2.09 (1.54, 2.85)	2.43 (1.72, 3.44)*
	Poor	99	337	1	1
Knowledge toward SHI	Good	72	216	0.70 (0.51, 0.97)	0.93 (0.63, 1.37)
	Poor	164	344	1	1
Presence of acute illness	Yes	61	262	0.40 (0.28, 0.55)	0.48 (0.32, 0.72)*
	No	175	298	1	1
Presence of chronic illness	Yes	173	447	0.69 (0.49, 0.99)	0.829 (0.55, 1.26)
	No	63	113	1	1
Family size	1–2	30	88	0.89 (0.54, 1.46)	0.99 (0.52, 1.87)
	3–4	130	271	1.25 (0.89, 1.76)	1.21 (0.81, 1.82)
	>4	74	193	1	1

AOR, Adjusted Odds Ratio; COR, Crude Odds Ratio; 1, Reference, ^*^Significant at P-value < 0.05.

## Discussion

The economy of a country is based on the total health of its population, which is measured by equitable and efficient health care (26). However, inadequate HCF continues to be a serious concern for Ethiopia's health-care system. Across 2011, Ethiopia's OOP health spending was 79.87%, compared to 62.2% in Sub-Saharan Africa. In 2011/2012, there was also a 34% budget gap for the delivery of health services. SHI is one of the primaries means of health finance used around the world to solve such a problem. The SHI Proclamation was adopted by Parliament in July 2010, but it has yet to be implemented due to a variety of obstacles (27), primarily owing to public sector civil workers' opposition (12, 23). In DCA, North-East Ethiopia, this study looked at the WTP for SHI and the factors that influence it among government employees.

In the current study, although the majority of participants (63.8%) had a good understanding of SHI, 54.8% had a negative attitude (perception) about it. Broadcast media (radio and television), followed by social media, were the primary sources of information for civil servants on SHI, accounting for 45.95 and 29.76%, respectively. However, this result was significantly lower than that of the research conducted in Bhaktapur Municipality, which found that 87.2% of participants were aware of SHI; insurance agents (47.3%) and female community health volunteers (28.6%) being the main sources of information (28). In contrast to this finding, a qualitative study conducted in Addis Ababa, Ethiopia, discovered that there was limited understanding of the concept and components of health insurance (29).

This study found that 30 and 8.2% of the participants, respectively, opted to pay second lowest and higher bids. The total magnitude of WTP for SHI was 29.6%, with age, positive view of SHI, acute illness, level of education, and net family income being the factors that were significantly linked with the WTP for SHI. This was slightly similar to the findings of other studies conducted in Debere Berhan, Ethiopia (27.8%) (30) and Northwest Ethiopia (32%) (19). This result was discovered to be higher than that of studies conducted in Arba Minch, Southern Ethiopia (5.9%) (31) and at St. Paul's Hospital Millennium Medical College, Addis Ababa, Ethiopia (17%) (32), but significantly lower than the findings of many other studies in Jimma, Southwest Ethiopia (90%) (13), Wolaita Sodo, South Ethiopia (74.4%) (15), Gondar, Northwest Ethiopia (62.0%) (11), Mekelle, Northern Ethiopia (85.3%) (23), Mujja, Ethiopia (37.6%) (33), Bahir Dar, Northwest Ethiopia (66.6%) (20), Mekelle, Northern Ethiopia (74.9%) (18), and public hospitals of Tigrai, Northern Ethiopia (35.5%) (34). When compared to other findings outside Ethiopia, such as in Akwa Ibom State, Nigeria (82%) (35), Sarawak, Malaysia (46.7%) (16), Bangladesh (67.5%) (36), Juba City, South Sudan (68%) (37), Public University in Malaysia (72.5%) (38), Pokhara Lekhnath, Nepal (51%) (39), Kampala, Uganda (91%) (40), and central Vietnam (71.6%) (41), the WTP for SHI in this study was very low. It was, however, much greater than a research conducted in Indonesia's Yogyakarta Province (1.67%) (42). As indicated, the WTP for SHI among civil servants in this study was extremely low, which could be related to the fact that inflation has a significant impact on civil servants whose salaries remain static (43). The discrepancy could also be attributable to differences in premium cut-off points, study duration, study subject selection, and sociodemographic status between regions and countries.

While the desire to receive free services during illness (43.7%) and supporting others (26.3%) were the primary reasons for the WTP for SHI, the main reasons for the unwillingness to pay were a preference for OOP (20.81%), a perception of a lack of laboratory tests (19.91%), and a lack of trust in the scheme (17%). It was also reported that people choose to pay for SHI in the hopes of receiving free health treatments (39). In another study, 66.5% of participants said it would help them save money on OOP expenses and in emergency medical circumstances (28). In fact, the theory of SHI is based on the uncertainty of illness (44).

When it came to the associated factors, age, a favorable attitude of SHI, acute illness, level of education, and net family income were the primary variables influencing the decision to pay for SHI. According to this study, respondents within the ages of 25–29 were more likely to pay for SHI than those over the age of 39. Many other research also found that age was inversely associated to the WTP for SHI (16, 23, 36, 45). On the other hand, the result of this study was in contrast to a research in Tigrai, Northern Ethiopia, which found that respondents above the age of 39 were more likely to be prepared to pay for SHI than respondents under the age of 24 (34). In Malaysia, younger employees (those under 30 years old) reported higher WTP (38). The drop in WTP for SHI with age may be attributed to the fact that as people get older, their supplemental income sources decrease, making them unable to afford the premium. However, it has also been discovered that older people are willing to pay a higher premium than younger people (46).

Educational status was found to be negatively linked with WTP for SHI in this study. A study conducted in Mekelle, Ethiopia, came to the same conclusion (23), but in contrast to other studies that found that schooling had a beneficial impact on WTP for SHI, Sarawak, Malaysia (16), Wolaita Sodo, Southern Ethiopia (15), Gondar, Northwest Ethiopia (11), Debere Berhan, Ethiopia (30), Yogyakarta Province, Indonesia (42), Iran (47), and a systematic review (45). But in contradiction to the current study, the more knowledge about SHI individuals has, the higher the WTP for SHI was observed (41). The disparity could be due to the fact that the public civil servants in this study had a negative attitude toward SHI. It could also be attributed to a lack of faith in the scheme. Trust in the scheme, on the other hand, has a positive effect on the decision to pay for it (20). The WTP for it was positively influenced by good perceptions of quality health care and the adequacy of SHI benefit packages (30).

According to this study, 59.4% of the respondents' homes had experienced acute illness, and those who had a family member with an acute illness in the past 6 months were less likely to have WTP for SHI than those who did not have such a condition. This was in line with the findings of a research conducted in Gondar, Ethiopia's northwest region (11). However, research conducted in Addis Ababa, Ethiopia, found the contrary (21). In the current study, the negative association between the occurrence of acute illness in the previous 6 months and the WTP for SHI could be due to bad health-care experiences. Because the existing health service is inadequately equipped and staffed, the existence of substandard healthcare service has been observed (27). The perceived quality of health-care services, on the other hand, was found to raise the WTP for SHI (18). It has also been reported that the ease with which past medical healthcare costs can be covered has a negative impact on the decision to pay for SHI (19). Alternatively, they may not have a history of financial difficulties in paying medical expenditures (18). That is, if the ill persons' healthcare costs are discovered to be too low, they may choose OOP. This will result in a negative decision to pay for the scheme, despite the fact that high-quality healthcare services may be available at the facilities (19). On the contrary, if there has been a history of high OOP healthcare costs, the plan's WTP may be favorable (32).

While income increased, so did the WTP for SHI, as also documented in other studies (11, 16, 23, 42, 45). In reality, as the monthly salary rises, so does the WTP for the scheme (11). If this is the case, however, poor people will be excluded unless they are subsidized. On the other side, when subsidized (poorer) groups receive fewer comprehensive benefits packages, equity will be jeopardized. As a result, it is difficult to manage, particularly for low income countries (48). So, the SHI program's ability to reach all communities in Africa has been hampered. As a result, relying entirely on the SHI scheme to achieve UHC may be unrealistic. Furthermore, excessively fragmented risk pools impede efforts to broaden insurance pools and encourage cross-subsidies (49).

Participants with a positive outlook were more likely to pay for SHI than those with a negative attitude. This result was consistent with the findings of other investigations (11, 20, 30, 47). However, the majority of the participants (54.8%) had a negative attitude toward SHI. The introduction of SHI may have been delayed as a result of such negative views. In fact, it has been claimed that SHI implementation has been postponed several times, owing to strong opposition from public employees (12). This problem could be rectified by making SHI membership mandatory. By pooling monies from mandatory funding sources, financial risks associated with illness can be distributed throughout the public servants (2); and allows everyone to get adequate health care when they need it without having to worry about user fees or OOP expenses (50).

### Practical and policy recommendations

Risk mix, mutual assistance, and broad coverage of groups are all advantages of SHI (51). However, the ability of the scheme to provide adequate financial risk protection is determined by the government's commitment to design the scheme as part of a national financing strategy (49). As a result, the EHIA is advised to set the premium as low as possible until the SHI is accepted. To improve the scheme's trustworthiness among civil servants, the agency should also expand the benefit packages. Moreover, it is commendable to start with voluntary civil servants and gradually make it mandatory.

### Strength

The study looked at a wide range of professionals in the public civil service pool. To infer appropriate information on the WTP for SHI and to summarize this interest to the broader public civil personnel, a large sample size was determined.

### Limitations

The study did not triangulate qualitative methods with a cross-sectional survey to delve deeper into the participants' perceptions of the SHI.

## Conclusion

The majority of the participants had a strong understanding of SHI but also a negative attitude about it. The size of the WTP for SHI among government employees was found to be minimal in this study (sub-optimal). The scheme's WTP was influenced by age, perception, net household income, the presence of acute illness, and educational status.

## Data availability statement

The original contributions presented in the study are included in the article/supplementary material, further inquiries can be directed to the corresponding author.

## Ethics statement

The study, involving human participants, was reviewed and approved by Wollo University's Medicine and Health Science College's Research, Community Service, and Graduate Coordinating Office. The patients/participants provided their written informed consent to participate in this study.

## Author contributions

EMA and FWF conceived and designed the study, conducted the study, analyzed and interpreted the data, contributed materials, and analysis tools or data. AMM and EMB conceived and designed the study, conducted the study, analyzed and interpreted the data, and wrote the article. All authors contributed to the article and approved the submitted version.

## Conflict of interest

The authors declare that the research was conducted in the absence of any commercial or financial relationships that could be construed as a potential conflict of interest.

## Publisher's note

All claims expressed in this article are solely those of the authors and do not necessarily represent those of their affiliated organizations, or those of the publisher, the editors and the reviewers. Any product that may be evaluated in this article, or claim that may be made by its manufacturer, is not guaranteed or endorsed by the publisher.
